# Morphologic response to chemotherapy containing bevacizumab in patients with colorectal liver metastases: A post hoc analysis of the WJOG4407G phase III study

**DOI:** 10.1097/MD.0000000000022060

**Published:** 2020-09-04

**Authors:** Ayumu Hosokawa, Kentaro Yamazaki, Chu Matsuda, Shinya Ueda, Hitoshi Kusaba, Shu Okamura, Masahiro Tsuda, Takao Tamura, Katsunori Shinozaki, Takahiro Tsushima, Takashi Tsuda, Tsuyoshi Shirakawa, Haruhiro Yamashita, Satoshi Morita, Shuichi Hironaka, Kei Muro

**Affiliations:** a Department of Clinical Oncology, University of Miyazaki Hospital, Miyazaki; b Division of Gastrointestinal Oncology, Shizuoka Cancer Center, Shizuoka; c Department of Surgery, Osaka General Medical Center, Osaka; d Department of Medical Oncology, Kindai University Nara Hospital, Nara; e Department of Hematology, Oncology and Cardiovascular Medicine, Kyushu University Hospital, Fukuoka; f Department of Surgery, Suita Municipal Hospital, Suita; g Department of Gastroenterological Oncology, Hyogo Cancer Center, Hyogo; h Department of Medical Oncology, Kindai University Faculty of Medicine, Osakasayama; i Division of Clinical Oncology, Hiroshima Prefectural Hospital, Hiroshima; j Department of Clinical Oncology, St Marianna University School of Medicine, Kawasaki; k Department of Chemotherapy, Miyazaki Prefectural Miyazaki Hospital, Miyazaki; l Department of Clinical Oncology, National Hospital Organization, Okayama Medical Center, Okayama; m Department of Biomedical Statistics and Bioinformatics, Kyoto University Graduate School of Medicine, Kyoto; n Department of Medical Oncology and Hematology, Oita University Faculty of Medicine, Yufu; o Department of Clinical Oncology, Aichi Cancer Center Hospital, Nagoya, Japan.

**Keywords:** bevacizumab, chemotherapy, colorectal cancer, liver metastases, morphologic response

## Abstract

The phase III West Japan Oncology Group (WJOG) 4407G study showed noninferiority of folinic acid, bolus/continuous fluorouracil, and irinotecan plus bevacizumab to modified folinic acid, bolus/continuous fluorouracil, and oxaliplatin 6 plus bevacizumab in progression-free survival (PFS) as first-line chemotherapy for patients with metastatic colorectal cancer. The aim of this study was to evaluate the predictive and prognostic value of morphologic response in patients with colorectal liver metastases (CLM) as a post hoc analysis of the WJOG4407G study.

Morphologic response was assessed by comparing contrast-enhanced computed tomography (CT) images at baseline and week 8. Three blinded radiologists evaluated CT images and classified their response as optimal, incomplete, or no response according to the morphologic criteria. Response evaluation criteria in solid tumors (RECIST) response, early tumor shrinkage (ETS), and depth of response (DpR) were also evaluated.

Among 395 patients who were eligible for efficacy analysis in the WJOG4407G study, 70 patients had liver-limited disease. We finally evaluated 55 of these patients. Optimal morphologic response was identified in 19 of 55 patients (34.5%). The median PFS was 10.7 months for patients with optimal response and 10.1 months in those with incomplete/no response (log-rank, *P* = .96). The median overall survival (OS) was 26.2 and 35.5 months, respectively (log-rank, *P* = .062). According to univariate analysis, morphologic response was not associated with PFS or OS, whereas RECIST response was significantly associated with both PFS and OS, with ETS and DpR being associated with significantly longer PFS.

Morphologic response might be neither a predictive nor a prognostic factor in patients with CLM undergoing chemotherapy containing bevacizumab, whereas RECIST response was significantly associated with both PFS and OS.

## 1 Introduction

In patients with metastatic colorectal cancer, FOLFIRI (folinic acid, bolus/continuous fluorouracil, and irinotecan) or FOLFOX (folinic acid, bolus/continuous fluorouracil, and oxaliplatin) plus bevacizumab are considered as standard first-line chemotherapy. The phase III WJOG (West Japan Oncology Group) 4407G study showed noninferiority of FOLFIRI plus bevacizumab to modified FOLFOX6 plus bevacizumab in progression-free survival as the first-line chemotherapy for patients with metastatic colorectal cancer (mCRC).^[1]^

Recently, with the advances in chemotherapy for advanced colorectal cancer, and particularly the development of molecular-targeted agents, analyses of predictive values by various image evaluation approaches have been conducted.

Early tumor shrinkage (ETS) is defined as the relative decrease in the sum of the longest diameters of target lesions from the baseline at the first evaluation (usually week 6 or 8). A cutoff value of ETS 20% or more was significantly correlated with longer progression-free survival (PFS) and overall survival (OS) in mCRC patients who received chemotherapy with antiepidermal growth factor receptor antibody.^[2]^ Depth of response (DpR), defined as the maximum tumor shrinkage in the sum of the longest diameters of target lesions, and ETS and DpR were reported to be highly associated with PFS and OS in mCRC patients treated with first-line chemotherapy plus bevacizumab.^[3]^

Morphologic changes on enhanced computed tomography (CT) are non-size-based and have been described when assessing tumor response to chemotherapy in patients with colorectal liver metastases (CLM). Morphological response criteria are based on the evaluation of tumor attenuation and margin ^[4]^ and several studies demonstrated that the morphologic response was associated with pathologic response ^[4,5]^ and survival outcomes ^[4–8]^ for patients with CLM undergoing chemotherapy with bevacizumab. However, these studies were retrospectively investigated at a single institution or at 2 institutions. The aim of this study was to evaluate the predictive and prognostic value of morphologic response to first-line chemotherapy containing bevacizumab in patients with CLM as a post-hoc analysis of the multicenter phase III WJOG4407G study.

## 2 Materials and methods

### 2.1 Patient population

We selected CLM patients enrolled in the phase III WJOG4407G study.^[1]^ Patients were randomly assigned to either FOLFIRI plus bevacizumab or modified FOLFOX6 plus bevacizumab with minimization stratified by institution, adjuvant chemotherapy, and liver-limited disease. Radiological assessments were repeated every 8 weeks.

### 2.2 Imaging analysis

**Figure 1:**
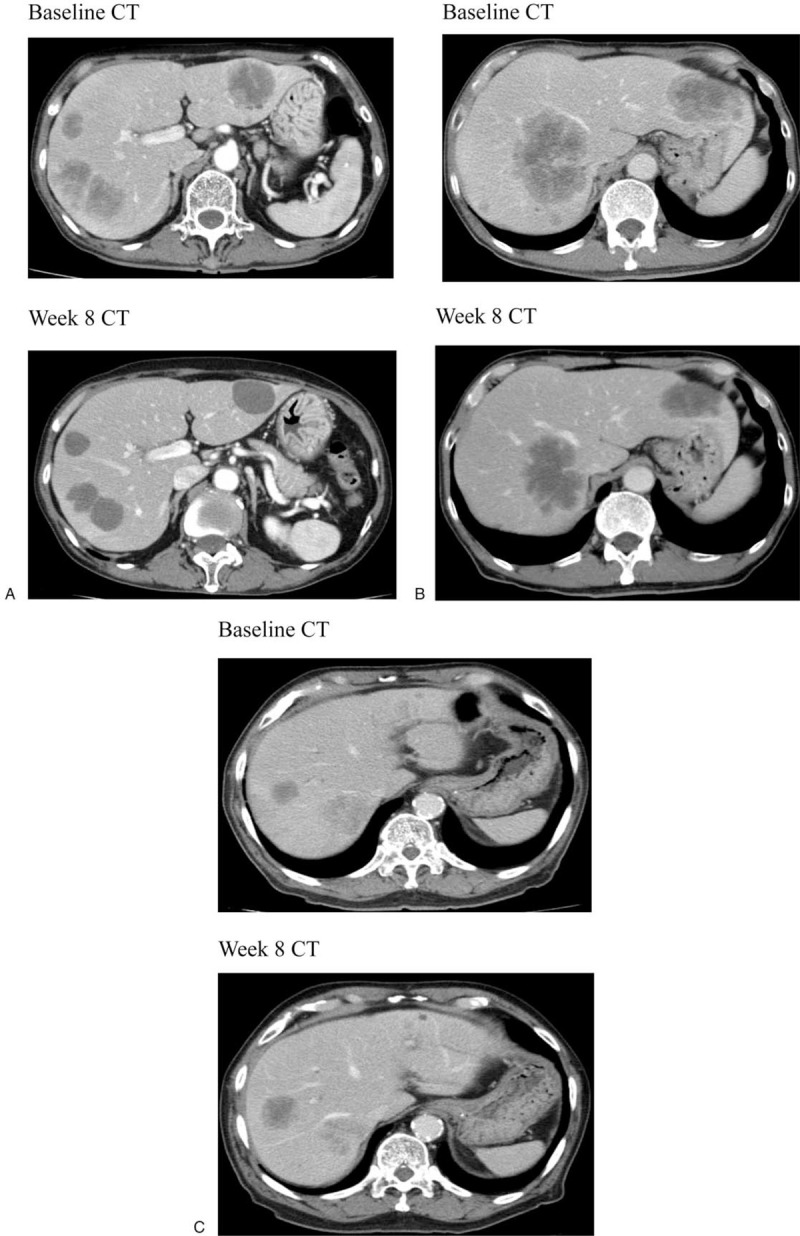
Optimal, incomplete, and no morphologic response after treatment. A, Optimal response. B, Incomplete response. C, No response.

Enhanced CT images from participating centers of the WJOG4407G study were collected. Morphologic response was assessed at 8 weeks compared with baseline CT. Three blinded radiologists evaluated CT images independently and classified responses as optimal, incomplete, or none according to the morphologic criteria.^[4]^ A group 1 metastasis had homogenous hypoattenuation with a thin, sharply defined-normal liver interface. A group 3 metastasis had heterogenous attenuation with a thick, poorly defined tumor-normal liver interface. A group 2 metastasis had morphology that did not qualify for either group 1 or 3 metastasis. Optimal response was defined as a change in morphology from group 3 or group 2 to group 1 after treatment. Incomplete response was defined as a change in morphology from group 3 to group 2, and no response was defined as the tumor not changing or increasing in morphology (Fig. 1). In discordant cases in morphologic response evaluation, the images were reviewed together by radiologists and a consensus resolution was reached.

Tumor responses, ETS, and DpR were also evaluated. Responses were evaluated according to Response Evaluation Criteria in Solid Tumors (RECIST) version 1.1. ETS was defined as a 20% or more decrease in the sum of the longest diameters of target lesions at 8 weeks. DpR was defined as the percentage of maximal tumor shrinkage in the sum of the longest diameters of target lesions at the nadir as compared with baseline values.

The protocol of the present study was approved by the ethics committees of all participating institutions. This study was registered in the University Hospital Medical Information Network (UMIN) Clinical Trials Registry, number UMIN000022171.

### 2.3 Statistical analysis

Categorical variables were compared using the *χ*^2^test or Fisher exact test, and continuous variables were compared using the Wilcoxon rank-sum test between the 2 groups. PFS was defined as the interval from the date of randomization to the date of confirmation of disease progression or death from any cause. OS was defined as the period from the date of randomization to the date of death from any cause. PFS and OS were calculated with the Kaplan–Meier method, and significant differences between survival curves were determined by the log-rank test. To identify predictive factors for survival, univariate analysis was performed using Cox proportional hazards model. All statistical analyses were performed with JMP version 14 (SAS Institute, Cary, NC), and *P* values of < .05 were considered to indicate statistical significance.

## 3 Results

### 3.1 Patients’ characteristics

Of 395 patients who were eligible for efficacy analysis in the WJOG4407G study, 70 patients had liver-limited disease. Enhanced CT images of 57 (81.4%) of 70 patients from 22 participating centers were collected. However, 2 patients were excluded from this analysis because their metastases became too small (less than 10 mm in diameter) to evaluate morphologic response after chemotherapy. The characteristics of the final patient cohort (n = 55) are shown in Table 1. The median age was 63 years (range, 35–75 years). All patients had a good Eastern Cooperative Oncology Group performance status. Fifty patients (91%) had multiple liver lesions. Twenty-six patients (47%) received modified FOLFOX6 plus bevacizumab and 29 (53%) received FOLFIRI plus bevacizumab as the first-line chemotherapy. Although FOLFIRI plus bevacizumab tended to have a higher frequency of solitary liver metastasis (*P* = .053), baseline characteristics were not statistically different between modified FOLFOX6 plus bevacizumab and FOLFIRI plus bevacizumab.

**Table 1:**
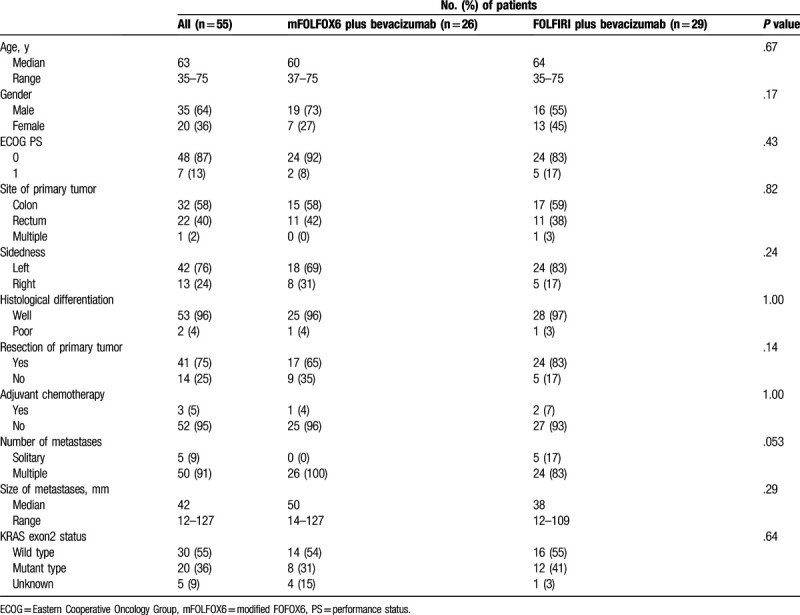
Patients’ characteristics (n = 55).

### 3.2 Efficacy

Efficacy parameters are summarized in Table 2. Among all patients, optimal response was observed in 34.5% according to morphologic response criteria. The best RECIST response observed was partial response (PR) in 60% of patients and stable disease (SD) or progressive disease (PD) in 40% of patients. RECIST response was not associated with morphologic response. Thirteen patients (39.4%) of PR and 6 patients (27.3%) of SD or PD by RECIST had optimal response (*P* = .35). ETS was observed in 58.2%, and the median DpR was 37.6% (range,ï¿1/2-10.4%-100%). The median PFS was 10.4 months and the median OS was 30.4 months in all patients. There were no statistically significant differences in efficacy parameters between modified FOLFOX6 plus bevacizumab and FOLFIRI plus bevacizumab.

**Table 2:**
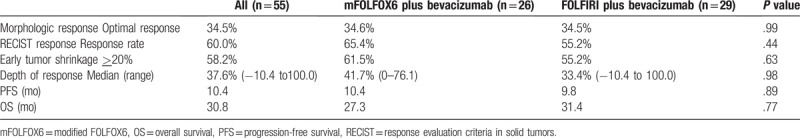
Efficacy by treatment arm.

**Figure 2:**
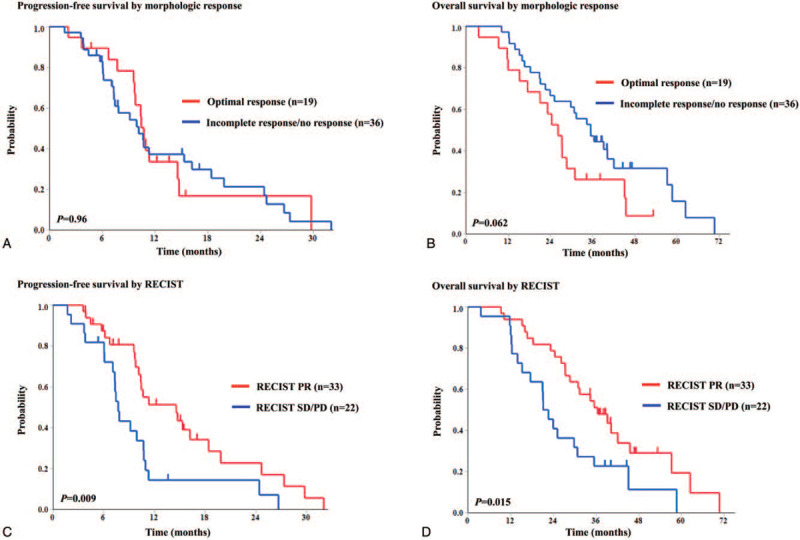
Kaplan–Meier curves for (A) progression-free survival by morphologic response, (B) progression-free survival by RECIST, (C) overall survival by morphologic response, and (D) overall survival by RECIST. RECIST = response evaluation criteria in solid tumors.

The median PFS by morphologic response was 10.7 months in patients with optimal response and 10.1 months in those with incomplete or no response (*P* = .96; Fig. 2A), while the median PFS by RECIST was 14.6 months in patients with PR and 7.7 months in patients with SD/PD (*P* = .009; Fig. 2B).

The median OS by morphologic response was 26.3 months in patients with optimal response and 35.5 months in those with incomplete or no response (*P* = .062; Fig. 2C), while the median OS by RECIST was 36.4 months in responders and 21.9 months in nonresponders (*P* = .015; Fig. 2D).

### 3.3 Predictive factors of PFS and prognostic factors of OS

Table 3 lists the results of univariate analysis of PFS and OS. Factors related to tumor shrinkage, RECIST response, ETS, and DpR (>38% vs < 38%) were significant predictors for PFS, however, optimal response had no predictive significance. Moreover, optimal response had no prognostic significance but RECIST response was the only prognostic factor of OS.

**Table 3:**
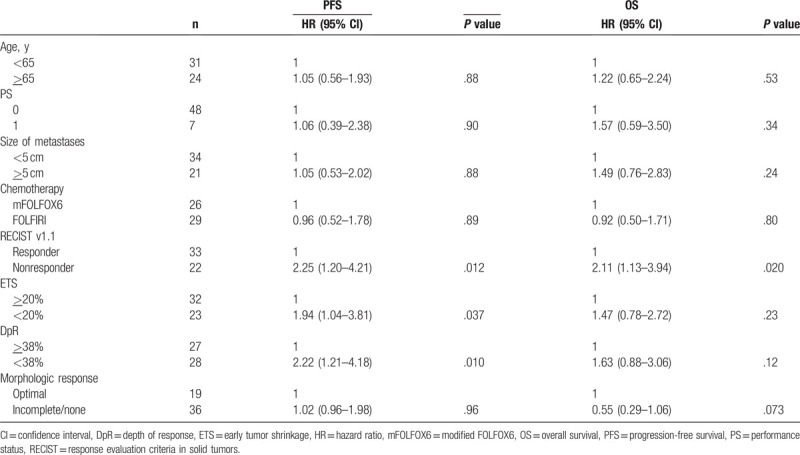
Univariate analysis of PFS and OS.

## 4 Discussion

Several studies have reported the predictive value of morphologic response in patients with CLM who were treated with fluorouracil-based chemotherapy.^[4–8]^ They included CLM patients that not only had extrahepatic disease ^[4–6,8]^ but that were also treated with fluorouracil-based chemotherapy with or without bevacizumab.^[5–8]^ In the present study, we evaluated the predictive and prognostic value of morphologic response to first-line chemotherapy containing bevacizumab in 55 patients with liver-limited mCRC as a post-hoc analysis of a phase III trial. Enhanced CT images were collected from 22 institutions where possible. Patients received either modified FOLFOX6 plus bevacizumab or FOLFIRI plus bevacizumab. According to the univariate analysis, morphologic response was not associated with PFS or OS, whereas RECIST response was significantly associated with both PFS and OS, with ETS and DpR being associated with significantly longer PFS. We could not show the usefulness of morphologic response to first-line chemotherapy containing bevacizumab in patients with CLM, whereas size-based response remains an important parameter of evaluation in treatment efficacy even in chemotherapy containing bevacizumab because RECIST response was significantly associated with both PFS and OS, with ETS and DpR being associated with significantly longer PFS in the present study.

A possible explanation for the lack of association with morphologic response and PFS or OS might be the post-hoc analysis. An enhanced CT imaging protocol was not specified and most of the patients were evaluated using single-phase enhanced CT imaging. Although a triple-phase enhanced CT protocol was rarely used in the present study, it was suggested to improve sensitivity by allowing assessment of early and delayed phases of tumor enhancement.^[4]^ In fact, the concordance rate of optimal response or incomplete/no response in the morphologic response as assessed by 3 radiologists was 82% (45/55); therefore, there were some cases in our study in which it was difficult to evaluate morphologic response precisely.

Morphologic criteria were reported to be strongly predictive of prolonged PFS in selected 142 patients with unresectable CLM in the NO16966 study,^[9]^ a phase III randomized trial that evaluated the efficacy and safety of first-line treatment with bevacizumab and oxaliplatin-based chemotherapy. In this study, morphologic response was assessed at first (week 6) and second (week 12) restaging, and an optimal morphologic response of 19% and 46%, respectively, was observed. Although this study included 82 patients with extrahepatic metastases, morphologic response at second restaging was associated with PFS compared with morphologic response at first restaging.^[10]^ It seems that standardization of enhanced CT imaging protocols and morphologic response at second restaging may be useful in examining the significance of morphologic response.

The present study has several limitations. Although this is a multicenter study including 22 institutions, it is a post hoc analysis and it could not include approximately 20% of the patients with liver-limited mCRC. Furthermore, in our study, the number of patients was limited due to the small population. Therefore, a prospective study of a large number of patients is recommended to assess the value of morphologic response to first-line chemotherapy containing bevacizumab in patients with CLM.

## 5 Conclusion

In summary, morphologic response might be neither a predictive nor a prognostic factor in patients with liver-limited mCRC undergoing chemotherapy containing bevacizumab, whereas RECIST response was significantly associated with both PFS and OS. Further evaluation will be needed to confirm the usefulness of morphologic response in patients with CLM treated with bevacizumab in a prospective study.

## Acknowledgments

The authors thank the patients and all the investigators who contributed to this study. The authors also thank the members of the WJOG Data Center, especially Dr Shinichiro Nakamura and Kaori Mori. They are grateful to the radiologists who evaluated the CT images (Dr Hideto Kawabe, Dr Gakuto Tomizawa, and Dr Norihito Naruto; University of Toyama). Finally, they thank H. Nikki March, PhD, from Edanz Group (www.edanzediting.com/ac) for editing a draft of this manuscript.

## Author contributions

**Conceptualization:** Ayumu Hosokawa, Kentaro Yamazaki.

**Data curation:** Chu Matsuda, Shinya Ueda, Hitoshi Kusaba, Shu Okamura, Masahiro Tsuda, Takao Tamura, Katsunori Shinozaki, Takahiro Tsushima, Takashi Tsuda, Tsuyoshi Shirakawa, Haruhiro Yamashita.

**Formal analysis:** Ayumu Hosokawa, Satoshi Morita.

**Supervision:** Shuichi Hironaka, Kei Muro.

**Writing – original draft:** Ayumu Hosokawa.

**Writing – review & editing:** Kentaro Yamazaki, Shuichi Hironaka.
